# Lysinuric protein intolerance: Unusual clinical manifestations in a compound heterozygote with a novel pathogenic variant

**DOI:** 10.1016/j.bbrep.2025.102229

**Published:** 2025-10-22

**Authors:** José R. Pascual López, Wilfred Wu, Laura Konczal

**Affiliations:** Center for Human Genetics, University Hospitals Cleveland Medical Center, 11100 Euclid Avenue, Cleveland, OH, 44106, USA

## Abstract

Lysinuric protein intolerance is an amino acid transport disorder that leads to episodic hyperammonemia especially in times of protein loading. We report a 10-year-old male with severe failure to thrive who presented to the hospital due to somnolence. The patient's overall appearance suggested that he was younger than his chronological age. He was admitted due to an ammonia level of 250 μmol/L that rose to 374 μmol/L on repeat testing. Mild transaminitis with AST and ALT in the 100–200 mg/dL range was noted. Plasma amino acids showed elevated glutamine, alanine, and ornithine, with diminished arginine. Urine organic acids were remarkable for elevated orotic acid. He was treated initially with D10 containing IV fluids, intralipids, and IV sodium benzoate/sodium phenylacetate and l-arginine. Once stable, he was converted to an oral ammonia scavenger-currently well controlled on sodium benzoate alone after not tolerating sodium phenyl glycerate. His diet was titrated to his meet his caloric and protein needs (with restriction) and supplementation with l-arginine, l-citrulline and l-lysine. The patient's hyperammonemia has since resolved and his glutamine has normalized. Molecular testing revealed two pathogenic variants in *SLC7A7,* confirming his diagnosis of lysinuric protein intolerance.

## Highlights

• Clinical variability underscores the need for individualized management.
• **c.1383_1384del (p.Ile461Metfs6):** novel frameshift variant not previously reported.
• **c.726G > A (p.Trp242):** known missense variant associated with severe LPI phenotypes.
• Emphasizes the importance of characterizing novel variants.

## 1 Introduction

The patient presented to an outside hospital two weeks prior to admission due to constipation and failure to thrive. He was managed conservatively, and he was discharged after two days with the recommendation to increase his protein intake due to his failure to thrive and a referral to medical genetics was placed. The patient returned before the genetics evaluation due to upper respiratory symptoms and lethargy with an otherwise unremarkable neurological exam.

## 2 Methods

### 2.1 Anthropometrics and somatotropic axis

**Fig. 1:**
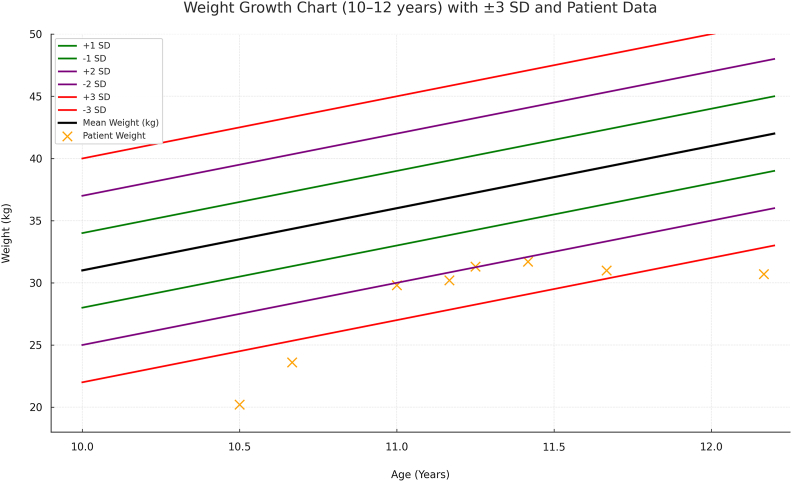
Weight growth chart with solid ±3 standard deviations.

**Fig. 2:**
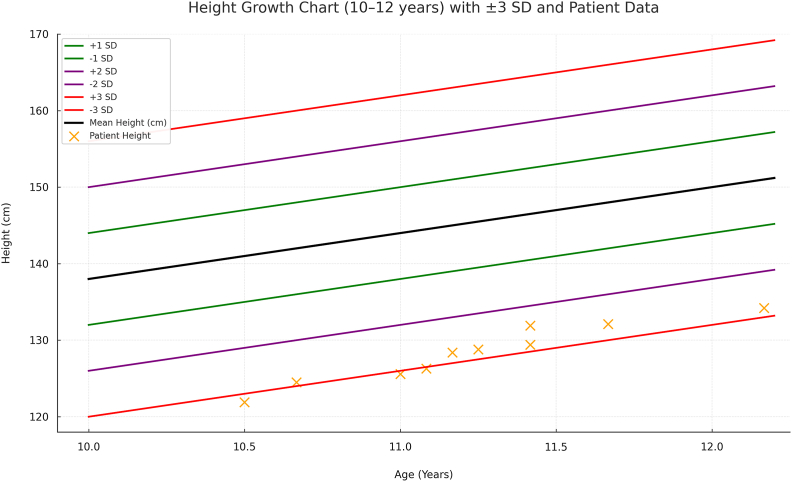
Height growth chart with solid ±3 standard deviations and patients data points.

The physical examination revealed a younger appearance than his chronological age due to short stature and low muscle bulk. His weight (20.2 kg) and height (122 cm) corresponded to Z-scores of −3.58 and −2.95, respectively. These values can be found in Fig. 1, Fig. 2 along with values obtained after hospital follow-up. A bone age assessment showed a delay of two standard deviations below the mean, resembling that of a 7-year-old. There were no signs of organomegaly. Laboratory evaluation for failure to thrive revealed low growth hormone (GH) activity, indicated by an Insulin-like Growth Factor Binding Protein 2 (IGFBP-3) level of 1.1 mcg/mL (reference range 2.4–8.4 mcg/mL).

### 2.2 Patient history and family history

The patient had an unremarkable prenatal and neonatal history. However, he had difficulties with growth for approximately nine years. His diet was diminished, and he ate no food rich in protein. We suspected that his hyperammonemia episode was triggered by this sudden increase in protein intake. This increase in intake was suggested as part of his management of his failure to thrive. There was no significant family history. The patient has a six-year-old sister who has no medical problems. Maternal ancestry is Russian, Irish, and English descent. Paternal ancestry is of German, Romanian, and Yugoslavian descent.

### 2.3 Management

Initial management for hyperammonemia consisted of IV sodium benzoate/sodium phenylacetate and l-arginine, D10–0.9%NS at 1.5 times maintenance and intralipids at 1 g/kg/day. Initially, his protein intake was restricted to 50 % of the daily recommended intake (DRI) based on his current weight. Considering his history of failure to thrive we also needed to increase the patient's calorie intake. This titration was difficult since the patient had a very noticeable aversion to protein-rich foods and preferred foods containing carbohydrates and fat. His neurological status improved with the above interventions. Due to his eating disorder, the patient required a gastrostomy tube. (G-Tube) since he could not meet the suggested daily protein requirements orally.

### 2.4 Initial evaluation

Our main clinical impression when the patient presented was a possible proximal urea cycle disorder (UCD) since he had a history of protein aversion. Also, he presented with hyperammonemia after an increase in protein load and in the setting of a viral illness which suggests a metabolic disease [1]. Initial metabolic workup consisted of plasma and urine amino acids, urine organic acids, urine orotic acid and plasma carnitine and acylcarnitine profile.

## 3 Results

### 3.1 Metabolic workup

**Fig. 3:**
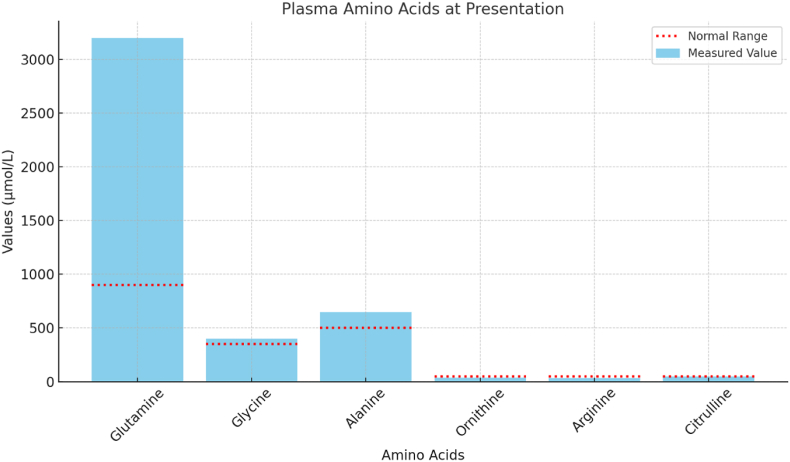
Plasma amino acids at presentation.

**Fig. 4:**
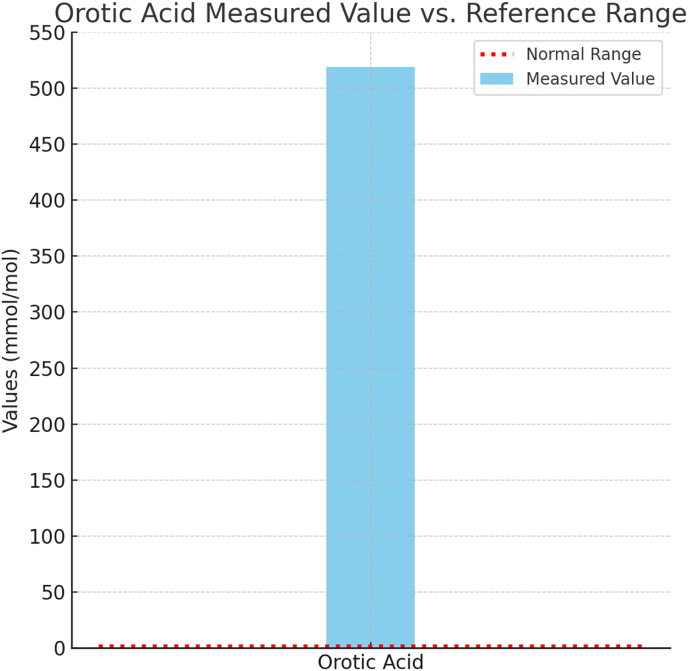
Orotic acid measured value vs. reference range.

Plasma carnitines were remarkable for low levels, which included total carnitine (12 μmol/L) and free carnitine (9 μmol/L). Plasma amino acids were remarkable for elevated glutamine with a value of 3180 μmol/L (460–750 μmol/) elevated glycine with a value of 452 μmol/L (150–550), elevated alanine of 661 μmol/L (230–550), diminished ornithine at 23.6 (30–130), arginine 16.4 μmol/L (50–150). Citrulline was normal with a value of 44 μmol/L. Urine orotic acid was elevated at 520/mole creatinine (<1.5 mmol/mol creatinine) (see Fig. 3, Fig. 4). Initial differential for hyperammonemia was a possible proximal UCD which included Ornithine Transcarbamylase (OTC) Deficiency and Carbamoyl Phosphate Synthetase I (CPS1) Deficiency, so empiric treatment was started before confirmatory molecular testing returned (including a protein restricted diet, ammonia scavengers, l-arginine and then l-citrulline with increase in calories)

### 3.2 Molecular testing and confirmation

**Fig. 5:**
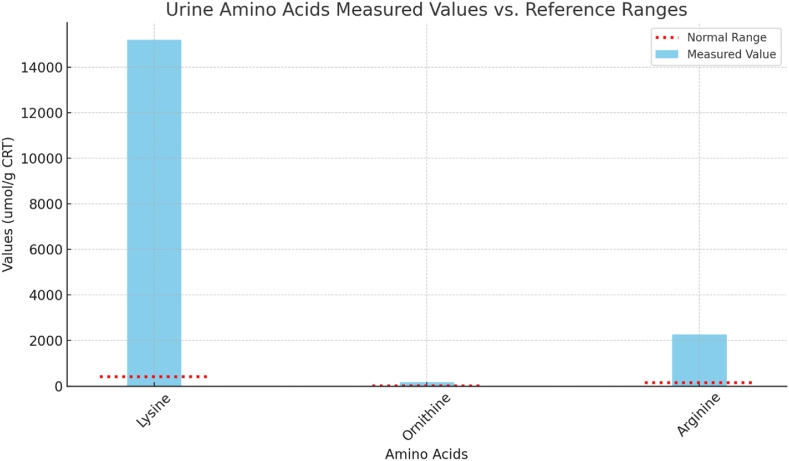
Urine amino acids measured values vs. reference ranges.

Molecular testing for hyperammonemia-related metabolic diseases identified two pathogenic variants in the *SLC7A7* gene: c.1383_1384del (p.Ile461Metfs6) and c.726G > A (p.Trp242). The c.1383_1384del (p.Ile461Metfs6) variant is a **novel frameshift mutation**, not previously reported in the literature or population variant databases (e.g., ClinVar, gnomAD). The second variant, c.726G > A (p.Trp242), is a **previously described missense mutation** associated with LPI in the **homozygous state**. However, to our knowledge, this is the **first documented case of this variant in a compound heterozygous context**, adding new insight into its role in disease when paired with a loss-of-function allele. [2]. The patient's symptoms of hyperammonemia, failure to thrive, and delayed bone age, combined with these genetic findings, led to a diagnosis of Lysinuric Protein Intolerance (LPI). Biochemical confirmation through urine amino acid analysis showed markedly elevated levels of lysine 15,213 μmol/g CRT (10–70), arginine 2278 μmol/g CRT (10–40), and ornithine 180 μmol/g CRT (10–30), corroborating the LPI diagnosis. These values can be found in Fig. 5.

## 4 Discussion

### 4.1 Hyperammonemia

In LPI, the typical plasma amino acid profile shows low citrulline, low arginine, and significantly elevated glutamine, resembling findings in proximal urea cycle disorders (UCDs). Hyperammonemia occurs due to decreased arginine and ornithine, which are crucial intermediates of the urea cycle. Arginine is a precursor for ornithine, essential for converting carbamoyl phosphate to citrulline and ultimately producing urea. Low ornithine levels lead to accumulation of carbamoyl phosphate, which is diverted into orotic acid synthesis, resulting in elevated orotic acid. Patients may unconsciously self-restrict protein in their diet to reduce symptoms, potentially delaying diagnosis as they remain asymptomatic for longer periods.

### 4.2 Prevalence

LPI is a rare autosomal recessive disorder with an estimated prevalence of as high as 1 in 60,000 births. It results from biallelic missense or nonsense mutations in the *SLC7A7* gene on chromosome 14q11.2, leading to a dysfunctional y + L amino acid transporter-1 (y + LAT-1). This transporter, comprising the light subunit y + LAT-1 and the heavy chain of surface antigen 4F2, is essential for cationic amino acid transport [3]. First described in Finnish patients in 1965, the *SLC7A7* gene was identified in 1998. Over 200 cases have been reported globally, primarily in Finland, Italy, and Japan, with some linked to consanguinity. Clinical presentations range from milder chronic symptoms to severe acute complications, including pulmonary, renal, and bone marrow involvement, even within the same family, highlighting the disease's heterogeneity [4].

### 4.3 Pathophysiology

The y + LAT-1 transporter facilitates the absorption and reabsorption of cationic amino acids (lysine, arginine, and ornithine) in the intestine and kidney, as well as in immune cells like monocytes and macrophages. This process involves the sodium-independent exchange of cationic amino acids for neutral amino acids (e.g., leucine, glutamine) and sodium [5]. This efflux from epithelial cells to the basolateral membrane by amino acids from inside the cells in exchange with neutral amino acids and sodium. In non-polarized cells, such as lymphocytes and macrophages, defects in the transporter cause intracellular amino acid imbalances, leading to excessive nitric oxide (NO) production via inducible NO synthase (iNOS) and promoting immune dysfunction. Additionally, an absence of the LAT transporter increases CCL5 expression, an inflammatory chemokine, potentially contributing to autoimmunity. While lysinuric toxicity in the kidney has been proposed as another mechanism, the precise links between these defects and the multi-organ manifestations of LPI remain unclear [5,6].

### 4.4 Clinical findings

#### 4.4.1 Liver findings

Clinical findings in LPI can be present at various ages ranging from infancy or childhood. The finding that is typically seen in childhood is poor growth since it is not necessarily evident in the neonatal period. One of the first findings that may be present is hepatosplenomegaly, which can be present since the newborn period and thought to occur due to macrophage dysfunction. Autopsies have revealed increased smooth endoplasmic reticulum and glycogen particles, fatty degeneration and cirrhosis which revealed chronic liver disease is part of the phenotype in LPI [7,8]. However, the lack of literature reporting the liver findings does not suggest a natural history of liver dysfunction in these patients.

### 4.5 Failure to thrive

As patients age poor growth becomes more apparent. This restriction has been thought to occur due to GH deficiency and abnormal bone remodeling. GH is thought to occur due to nutritional deficiencies and low arginine, which stimulates GH secretion [9]. However, growth restriction has also been observed even when nutritional deficiencies are corrected. Abnormal bone remodeling is thought to occur due to subconscious dietary protein restriction, lysine and GH deficiencies. These deficiencies lead to defective matrix protein synthesis and impaired capacity for maintenance of normal bone remodeling.

### 4.6 Lung findings

LPI patients can also present with lungs disease secondary to pulmonary alveolar proteinosis (PAP) or lung fibrosis. PAP is a rare lung disease involving surfactant accumulation within the alveoli resulting from decreased clearance. In general, it often presents as a secondary process to obstructive processes and pulmonary hypertension. Diagnosis is confirmed via bronchoscopy with bronchoalveolar lavage (BAL), and without treatment, such as lung transplantation, it can be fatal in childhood. Lung fibrosis has also been documented and in some cases suspected to be independent from PAP due to imaging findings of fibrosis with BAL that exclude PAP. It is hypothesized that increased concentrations of cationic amino acids in alveolar lining can lead to exaggerated inflammation in the alveolar spaces [10,11]. Our patient had a chest CT scan for screening which was negative.

### 4.7 Hemophagocytic lymphohistiocytosis (HLH)

There have been multiple cases of patients with LPI that have been diagnosed with hemophagocytic lymphohistiocytosis [[12], [13], [14]]. HLH is diagnosed by presence of fever, splenomegaly and mainly consists of infiltration of several organs by activated lymphocytes and macrophages.

Bone marrow biopsy can be remarkable for hypercellularity, macrophages phagocytosing erythrocytes and leukocytes among other findings. However typical bone marrow biopsy findings are not necessary to diagnose HLH. Bone marrow failure is also thought to occur.

### 4.8 Renal findings

Renal manifestations in patients with LPI typically presents in childhood and most commonly include hematuria, proximal tubular dysfunction leading to chronic kidney disease (CKD) and End Stage Renal Disease (ESRD). The histological findings are nonspecific and include glomerular amyloidosis, mild mesangial sclerosis, focal glomerulosclerosis, diffuse glomerulonephritis, glomerular basal membrane thickening with wire loops among others [15,16]. Some patients with LPI have also been found to have SLE after meeting the following criteria: serositis, renal, hematological, immunological disorders, positive anti-nuclear antibodies. These reports of SLE are other clinical examples that propose immune dysfunction by an unclear mechanism [17,18].

## 5 Long term treatment

**Fig. 6:**
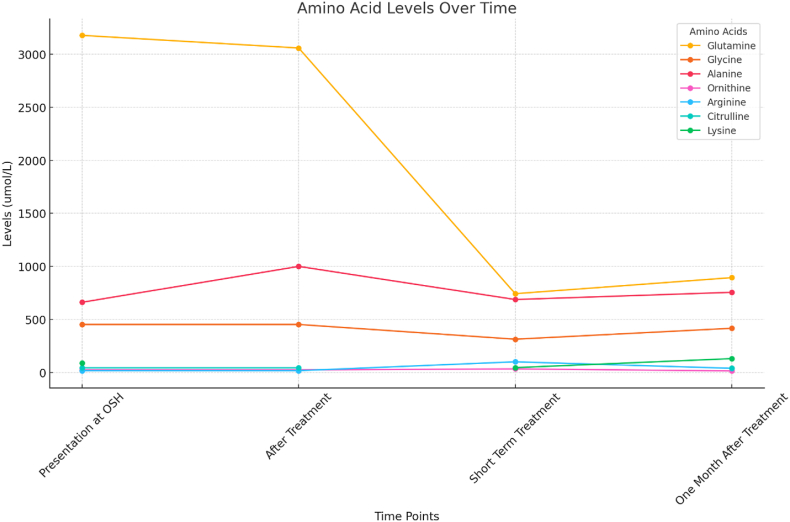
Amino acid levels over time.

The patient is managed with a restriction of total protein intake to **35**–**45 g per day**, with approximately **20 g** coming from intact protein sources, while the remainder is provided through a specialized low-nitrogen amino acids medical formula. To support metabolic control and nutritional needs, he is also on sodium benzoate (181 mg/kg/day), l-arginine (516 mg/kg/day), l-carnitine (16 mg/kg/day), l-lysine (29 mg/kg/day), and l-citrulline (96 mg/kg/day). This comprehensive regimen aims to optimize metabolic function, suppress catabolism and prevent complications associated with LPI. He has been screened for renal disease, PAP and HLH. All of the screening has been negative. The patient has improved clinically and has not had another episode of hyperammonemia. Repeat amino acids suggest that his average ammonia levels have been decreasing as evidenced by a decrease in his glutamine and glycine levels (see Fig. 6).

## 6 Conclusion

This case contributes novel genetic and clinical insights into Lysinuric Protein Intolerance (LPI), while also illustrating the disorder's significant phenotypic heterogeneity. The patient was found to be compound heterozygous for two pathogenic *SLC7A7* variants: a previously unreported frameshift mutation, c.1383_1384del (p.Ile461Metfs6), predicted to result in nonsense-mediated decay, and the c.726G > A (p.Trp242) missense variant, which has been previously reported only in the homozygous state. To our knowledge, this represents the first documented case of the p.Trp242 variant in a compound heterozygous context. The patient's biochemical profile—including markedly elevated urinary lysine, arginine, and ornithine—is diagnostic of LPI. However, he notably lacks several commonly reported complications such as pulmonary alveolar proteinosis (PAP), hemophagocytic lymphohistiocytosis (HLH), renal involvement, or hepatosplenomegaly. This phenotypic heterogeneity may reflect the influence of specific genotype combinations on disease expression and highlights the need for continued reporting of variant-level data. These findings highlight the value of genetic testing in identifying pathogenic variants that enhance our understanding of genotype-phenotype correlations, even in cases where biochemical testing has already confirmed the diagnosis.

## CRediT authorship contribution statement

**José R. Pascual López:** Conceptualization. **Wilfred Wu:** Supervision. **Laura Konczal:** Supervision.

## Genetic testing methods

Genomic analysis was performed using a clinically validated next-generation sequencing (NGS) assay. Genomic DNA was extracted from peripheral blood and enriched for target regions using a hybridization-based capture protocol. Sequencing was conducted on an Illumina platform, with alignment to the human genome reference build GRCh37.

All targeted coding exons and at least 20 base pairs of flanking intronic sequence were sequenced to a minimum depth of 50 × , with supplemental analysis applied where necessary to ensure full coverage. Variants were identified using a custom bioinformatics pipeline and interpreted according to ACMG guidelines.

Copy number variants (CNVs), including single- and multi-exon deletions and duplications, were detected using an in-house algorithm based on comparative read-depth analysis. Orthogonal confirmation using MLPA, MLPA-seq, or long-read sequencing (e.g., PacBio SMRT sequencing) was performed when indicated.

This assay is validated to detect >99 % of single nucleotide variants (SNVs) and small insertions/deletions (indels <15bp) in the targeted regions. RNA sequencing and long-range PCR were applied in select scenarios to resolve variant phasing, disambiguate pseudogene regions, or assess splicing effects.

## Ethical compliance

This study was conducted in full compliance with ethical standards for research involving human subjects. All procedures were performed in accordance with the ethical principles outlined in the Declaration of Helsinki. The manuscript adheres to the Recommendations for the Conduct, Reporting, Editing, and Publication of Scholarly Work in Medical Journals, including appropriate and accurate use of the terms sex and gender, and consideration of representative human populations with respect to sex, age, and ethnicity. Informed consent was obtained for all human subjects and/or biological samples included in this study, and participants’ privacy rights were fully respected throughout.

## Funding

This research did not receive any specific grant from funding agencies in the public, commercial, or not-for-profit sectors. All clinical evaluations and patient care were conducted as part of standard medical practice during the authors’ routine responsibilities—by Dr. Pascual López in his role as a resident physician, and by Drs. Konczal and Wu as attending physicians—at our academic medical center. All work was performed in accordance with institutional policies and ethical standards.

## Declaration of competing interest

“The authors declare that they have no known competing financial interests or personal relationships that could have appeared to influence the work reported in this paper."

## Data Availability

Data will be made available on request.
